# Advanced Strategies for Articular Cartilage Defect Repair

**DOI:** 10.3390/ma6020637

**Published:** 2013-02-22

**Authors:** Amos Matsiko, Tanya J. Levingstone, Fergal J. O’Brien

**Affiliations:** 1 Royal College of Surgeons in Ireland, 123 St. Stephen’s Green, Dublin 2, Ireland; E-Mails: amosmatsiko@rcsi.ie (A.M.); tanyalevingstone@rcsi.ie (T.J.L.); 2 Trinity Centre for Bioengineering, Trinity College Dublin, Dublin 2, Ireland

**Keywords:** articular cartilage, biomaterials, chondrogenesis, scaffolds, tissue engineering

## Abstract

Articular cartilage is a unique tissue owing to its ability to withstand repetitive compressive stress throughout an individual’s lifetime. However, its major limitation is the inability to heal even the most minor injuries. There still remains an inherent lack of strategies that stimulate hyaline-like articular cartilage growth with appropriate functional properties. Recent scientific advances in tissue engineering have made significant steps towards development of constructs for articular cartilage repair. In particular, research has shown the potential of biomaterial physico-chemical properties significantly influencing the proliferation, differentiation and matrix deposition by progenitor cells. Accordingly, this highlights the potential of using such properties to direct the lineage towards which such cells follow. Moreover, the use of soluble growth factors to enhance the bioactivity and regenerative capacity of biomaterials has recently been adopted by researchers in the field of tissue engineering. In addition, gene therapy is a growing area that has found noteworthy use in tissue engineering partly due to the potential to overcome some drawbacks associated with current growth factor delivery systems. In this context, such advanced strategies in biomaterial science, cell-based and growth factor-based therapies that have been employed in the restoration and repair of damaged articular cartilage will be the focus of this review article.

## 1. Articular Cartilage Structure and Function

In order to develop biomaterials for articular cartilage repair, it is important to understand the structure of the native tissue. Articular cartilage is mainly composed of hyaline cartilage which is found on articulating surfaces of bones in diarthroidal joints. It is a specialized connective tissue that provides a surface for frictionless movement of opposing bones in the joint. Hyaline cartilage is also found on the ventral ends of ribs and is distinguished from the other forms of cartilage by the high content of collagen type II and rich proteoglycan matrix synthesized by chondrocytes. This surface acts as a shock absorber for the loads experienced due to movement. Together with synovial fluid, this tissue provides lubrication and pain free motion with wear resistance over the course of an individual’s lifetime [1].

Articular cartilage forms a thin layer of tissue, varying in thickness depending on the location in the body; however, it is fully adapted to withstand compression forces. In humans, the articular cartilage can range from 1 to 4 mm depending on the joint [2]. This tissue is able to deform in order to increase the total surface area for contact thereby reducing the overall stress. Stress relaxation of articular cartilage, indicative of its visco-elastic properties, further demonstrates its unique function in resisting damage from applied loads [3]. Furthermore, this tissue is also capable of counteracting compression by pressurization of the interstitial fluid with more than 95% of the load carried by fluid [4]. The function of articular cartilage in generating resistance against compression and shear forces is dependent on the specific arrangement of its extracellular matrix macromolecules. In particular, the collagen fiber arrangement and orientation greatly dictate the amount and direction of deformation due to applied stress [5].

**Figure 1:**
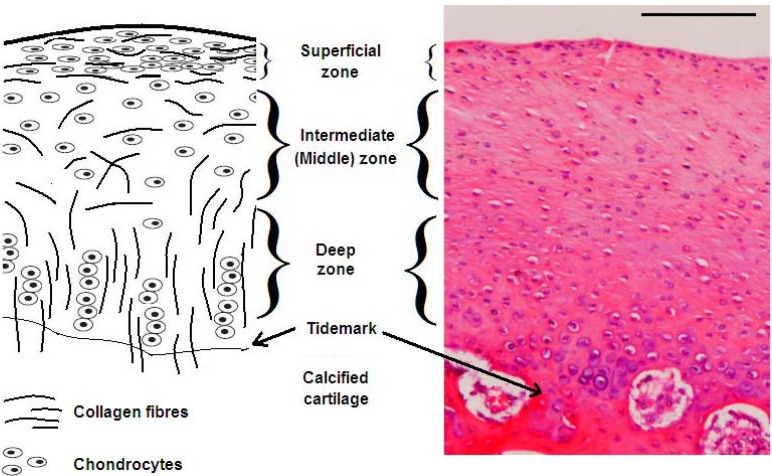
The structural organization of articular cartilage from the superficial zone to the deep zone and the calcified cartilage. The illustration on the left shows collagen fibers and chondrocytes of articular cartilage demonstrating the difference in their orientation and shape within the different zones. The histological image (haemotoxylin and eosin (H&E) staining) on the right is taken from the femoral condyle of a rabbit knee joint and demonstrates the distribution of chondrocytes within the different zones (scale bar represents 100 µm).

Articular cartilage is further organized into four zones: superficial, intermediate (middle), deep and calcified cartilage zones (Figure 1). Within the superficial zone, chondrocytes appear flat in shape at close proximity to each other and the collagen fibers are aligned parallel to the articular surface. In the intermediate zone, chondrocytes are oblique in shape and collagen fibers are randomly organized in different directions. Deep zone cartilage however, is characterized by spherical chondrocytes that are aligned in columns and the collagen fibers are perpendicular to the articular surface. The collagen fibers in the deep zone penetrate through the tidemark into the calcified cartilage to provide structural stability for articular cartilage on the subchondral bone [6].

## 2. Articular Cartilage Damage

Degradation of articular cartilage can arise from trauma, disease or continual mechanical loading. There are three main types of cartilage injury: superficial matrix disruption, partial thickness defects and full thickness defects. Superficial matrix disruption arises from blunt trauma whereby the ECM is damaged but viable chondrocytes aggregate into clusters and are capable of synthesizing new matrix [7]. Partial thickness defects disrupt the cartilage surface but do not extend into the subchondral bone. These defects are unable to self-repair unlike superficial matrix disruption [8]. Full thickness defects arise from damage that penetrates deep into the subchondral bone [1]. These defects can elicit a repair response due to access to the marrow cells; however, they are typically filled with fibrocartilage. This type of repair tissue is much weaker than hyaline cartilage and displays poor long-term performance due to poor compressive strength and durability and gradually leads to degeneration [9].

Traditional cartilage repair techniques attempt to reduce pain as well as restore functionality to tissue. The success of these techniques depends on their long-term performance as well as the similarity of the repair tissue to native articular cartilage in terms of the composition and mechanical properties [6]. Regeneration will not occur without access to cells such as progenitor cells or chondrocytes that can undergo chondrogenesis and synthesize *de novo* tissue. With this in mind, a number of repair strategies have been developed. Current clinical treatment techniques include Pridie drilling, microfracture, mosaicplasty and autologous chondrocyte implantation (ACI) [9,10,11,12]. These will be discussed in more detail below.

## 3. Cartilage Treatment Strategies: Current State of the Art

In relation to cartilage treatment strategies, Plewes highlighted in 1940 that, “further observations of the aetiology and treatment should therefore be of value not only with a view to ascertaining the best methods of treatment but also of preventing this crippling condition” [13]. In this regard, articular cartilage defects are classified according to their depth and width [14,15]. Healing of defects as a result of mechanical disruption of the chondral tissue limited to the articular surface differs from the healing response as a result of mechanical disruption affecting both the articular surface and subchondral bone,* i.e.*, an osteochondral defect [16]. Consequently, the choice of a clinical repair technique depends largely on the classification of the defects and whether the patient demand requires a palliative, reparative or restorative approach. Evidence-based decision algorithms are adopted to determine which approach to take depending on factors such as age, lesion size and activity of the patient [17,18].

### 3.1. Debridement and Lavage

Patients with chondral lesions of less than 2 cm^2^ diameter have been successful candidates for primary treatment options such debridement and lavage. This approach involves elimination of loose cartilage fragments within the joint. This technique is generally reserved for lower demand older patients with limited symptoms who would have difficulty with restricted post-operative mobility [19,20]. Although it is considered as a palliative treatment option, relief from pain and improvement in quality of life is generally quick with immediate full weight bearing and unrestricted activities [19].

### 3.2. Microfracture

Microfracture is adopted as a secondary treatment option for chondral lesions of less than 2–3 cm^2^ in diameter. However, unlike debridement and lavage, microfracture is a reparative strategy and is generally reserved for patients with moderate demands [19]. Microfracture involves drilling holes of approximately 0.5–1 mm diameter through the articular cartilage tissue and into the bone marrow cavity to allow access to bone marrow permitting progenitor cell recruitment to the injured site subsequently promoting defect repair [21,22]. However, continuous passive motion with limited weight bearing activities post-operatively is required to permit good healing response. Microfracture has been widely employed clinically and has been shown to permit effective short term functional improvement in joint function [23]. Medium to long term follow up studies have revealed limited hyaline-like cartilage tissue formation [24,25] in comparison to other techniques such as autologous chondrocyte implantation. Over time, the initially synthesized hyaline-like cartilage becomes more fibrous and deteriorates as a result of sub-optimal biomechanical and visco-elastic properties of the repair tissue. Inevitably, this repair tissue is prone to failure and a return of symptoms and thus this treatment option serves only to delay the eventual requirement for joint replacement [26,27]. A new approach is emerging that involves the combination of microfracture and a scaffold in order to enhance access to the bone marrow cavity. Erggelet* et al.* (2009) [28] employed a poly-glycolic acid (PGA) scaffold and hyaluronic acid to cover micro-fractured full-thickness articular cartilage defects within a sheep model. Compared to the microfracture-only controls which led to fibrocartilage formation, the combination of the cell-free scaffolds and microfracture facilitated enhanced cartilaginous repair tissue with evidence of collagen type II within the defects. Bone marrow aspirates have widely been used clinically for tissue repair. In particular, the use of concentrated bone marrow in combination with a scaffold and microfracture has been shown to enhance the regeneration of hyaline-like cartilage formation within a defect [29].

### 3.3. Autografts

Cell and tissue transplantation are generally reserved for patients with lesions larger than 2 cm^2^ diameter as secondary treatment options. These techniques are carried out on intermediate to high demand patients following failure as a result of microfracture or debridement to adequately solve the underlying problem. Treatment strategies relying on chondral and osteochondral autograft transplantation have been employed clinically. Autografts are reserved for small to medium chondral and osteochondral defects (up to 3 cm^2^ diameter) and on high-demand patients of an older age than those that would be treated with other procedures. Larger defects can be treated with other procedures. Larger defects can be treated using allograft tissue or mosaicplasty whereby a number of cartilage tissue plugs are extracted from a non-weight bearing region of the joint and implanted onto the defect site [19,30]. Major strengths of graft tissue are that the procedure can be performed arthroscopically as well as the fact that the defect is filled with healthy native cartilage. The main limitation of this procedure is donor site morbidity, limited lateral integration as well as joint incongruity as a result of multiple plugs [19,31].

## 4. Advances in Articular Cartilage Repair Using a Tissue Engineering Approach

The poor long-term outcome of conventional treatment methods used clinically demonstrates that there still remains an inherent need for alternative approaches in cartilage defect repair. Tissue engineering has shown promise in the repair of defects within cartilage tissue [32,33]. Although the rapidly growing field of tissue engineering has received a lot of attention since the late 20th century, the process of manipulation of tissue through grafting to restore or repair tissue has been carried out for many centuries. More recently, in March 1999, *Time Magazine* published an article entitled, “*How to build a body part*” by Josh Fishman [34]. It delved into the emerging field of tissue engineering. As the author highlights, “*yet the era of grow-your-own organs is already upon us, as researchers have sidestepped the stem-cell controversy by making clever use of ordinary cells … a teenager born without half of his chest wall is growing a new cage of bone and cartilage within his chest cavity”*. This article received a great deal of attention and the following year a cover story of the May 2000 issue of *Time Magazine* included predictions relating to future careers with great promise, listing tissue engineering as the top projected career [35]. This brought significant focus to the nascent field and was possibly one of the factors which contributed to an increase by international funding bodies of investment towards the field of tissue engineering thus resulting in an exponential expansion in research in the area from the year 2000 onwards.

**Figure 2:**
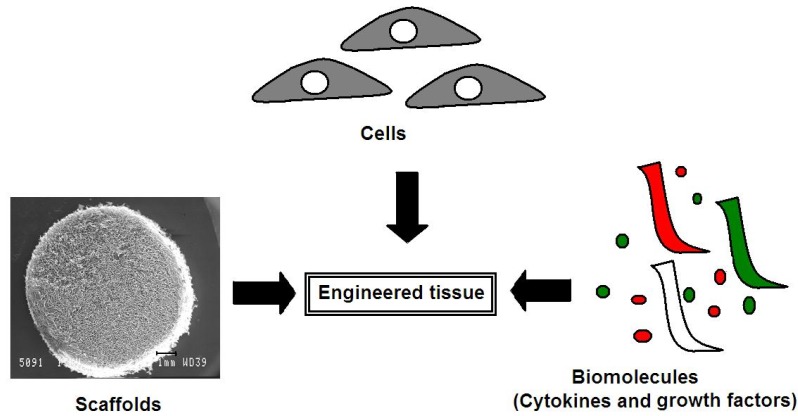
An illustration of the tissue engineering triad comprised of cells, scaffolds and biological signal molecules. (Scaffold scanning electron microscope (SEM) image reprinted with permission from [38]. Copyright 2012 Elsevier).

Tissue engineering has been adopted as a means to develop biocompatible substitutes that provide the appropriate biological cues to stimulate tissue regeneration with an eventual goal of improving or restoring tissue function. It is based on close ties between biomaterial science and cell biology and involves the interaction between three key aspects for successful restoration or regeneration of damaged tissue. This triad is comprised of cells, scaffolds and biological signal molecules (Figure 2). The cell source is fundamental in order to guarantee maintenance of the desired phenotype and synthesis of de novo tissue [36]. The scaffolds are required to provide the “template” for the cells to lay down synthesized matrix or potentially provide biological cues for modulating cell behavior such as proliferation, migration and differentiation. The biological signaling molecules are required to stimulate progenitor cell recruitment, differentiation as well as directing desired tissue synthesis [37]. This review will cover these governing areas of tissue engineering and highlight some of the recent advances in this rapidly growing field with specific application to cartilage defect repair.

### 4.1. Cells for Cartilage Defect Repair

The selection of a suitable cell type is fundamental in tissue engineering (TE) markedly affecting the desired repair tissue. A wide range of cell types have been adopted in cartilage TE applications. Whilst chondrocytes, the native cell type in cartilage tissue, are an obvious choice of cells, some drawbacks associated with their use* in vitro* have prompted researchers to look for alternative sources. Stem cells and progenitor cells derived from a range of sources have been widely investigated and show significant promise in cartilage repair.

#### 4.1.1. Chondrocytes

Articular cartilage possesses only one cell type; chondrocytes, which are embedded within their own rich extracellular matrix. The density of chondrocytes within articular cartilage is approximately 10,000 cells per mm^3^, which equates to 1.7% volume of tissue [39]. Taking this into context, it suggests that over 98% of articular cartilage is occupied by the extracellular matrix (ECM) which is modulated by the single cell type. This highlights the significance of using chondrocytes as viable cell sources for defect repair. *In vitro* studies utilizing chondrocytes have shown the potential of these cells in maintaining a chondrogenic phenotype when cultured in three-dimensional environments [40,41]. The ability of chondrocytes to synthesize cartilage-like matrix* in vitro* demonstrates their potential and regenerative capacity. Indeed chondrocytes have also been adopted clinically for the repair of chondral lesions in autologous chondrocyte implantation.

##### Autologous Chondrocyte Implantation (ACI)

In ACI, cells are isolated from non-weight bearing regions of a joint such as the lateral trochlear ridge [42]. The cells are expanded* in vitro* and transplanted to a defect and covered with a sutured periosteal flap. ACI has been used clinically since 1987 when it was initially introduced by Mats Brittberg in Sweden for the management of full thickness chondral defects [11]. A number of studies have demonstrated that the repair tissue as result of ACI is more durable and biomechanically similar to native hyaline-like cartilage tissue with better long term outcomes than microfracture [11,26,27]. The main drawbacks of ACI however, are the two-stage procedure required, the prolonged period of time required to expand sufficient numbers of chondrocytes* in vitro*, and the poor retention and dedifferentiation of cells following implantation in the clinic. The requirement of prolonged restrictions on weight-bearing through continuous passive motion post-operatively is another major drawback of ACI. In addition, a large open arthrotomy is associated with this procedure. Periosteal hypertrophy, ablation and loss of implanted cells from the defect site have also been identified as limitations of ACI that can lead to the requirement for additional surgery [19,43,44,45].

Consensus has yet to be reached on the clinical efficacy of ACI in comparison to microfracture. A number of factors come into play in the assessment of clinical outcomes. A study comparing ACI and mosaicplasty showed that athletes with chondral defects that were treated with mosaicplasty returned to active sports more quickly than those that were treated with ACI. However, the durability and clinical improvement was higher with ACI after a 42-month follow-up [46].

##### Collagen-Covered Autologous Chondrocyte Implantation (CACI)

A range of complications are associated with the use of a periosteal flap in ACI procedures, including graft delamination, potential ectopic calcification of the periosteal patch, and graft hypertrophy which can lead to “catching” of the knee joint. There has thus been a move towards the use of an alternative membrane to seal the cartilage defect. A range of different materials have been used, however, in the majority of clinical cases collagen-membranes have been used. This procedure is termed Collagen-Covered Autologous Chondrocyte Implantation (CACI) or Autologous Matrix-induced chondrogenesis or AMIC and marketed as Chondro-Gide^®^ or Bio-Gide^®^ by Geistlich Biomaterials (Wolhusen, Switzerland). Gooding* et al.* (2006) [47] carried out a comparative clinical and functional assessment comparing ACI with a periosteal cover (ACI-P) to an ACI with a type I/III collagen membrane (ACI-C) in 68 patients. No significant difference was found between results at one year post surgery. However, greater than 36.4% of ACI-P grafts showed signs of hypertrophy compared to none of the ACI-C grafts. Based on the study outcomes, Gooding recommended the use of ACI-C over ACI-P. A range of other clinical studies have also demonstrated the effectiveness of this technique [48,49,50,51,52].

##### Matrix-Induced Autologous Chondrocyte Implantation (MACI)

MACI has been developed as a second generation approach combining chondrocytes and a scaffold (matrix) [12,53,54,55,56]. The use of a scaffold provides a structure that facilitates chondrocyte adhesion, expansion whilst maintaining a chondrocytic phenotype limiting dedifferentiation which has been observed in two-dimensional systems [57]. Indeed, Zheng* et al.* (2007) [54] found that 75% of biopsies from a group of 11 patients at 6 months supported chondrocyte differentiation with resulting hyaline-like cartilage formation. A 5 year follow up after treatment with MACI showed that 8 out of 11 patients rated the function of their treated knees as “much better” or “better” than before treatment [10]. Another study carried out a 2–5 year follow-up assessment and showed that the over 91% of patients developed improved cartilage tissue on treated knees according to the International Knee Documentation Committee (IKDC) [58]. Although MACI has shown good healing response with better repair than ACI, a major drawback of this approach is the inability to treat large chondral defects as well as the inherent donor site morbidity due to chondrocyte harvest from cartilage tissue [59].

##### Drawbacks Associated with the Use of Chondrocytes in Cartilage Defect Repair

Whilst chondrocytes conceivably show great promise in the long term repair of lesions within articular cartilage, inherently, they possess some drawbacks that hinder their use in TE. Cell-based procedures utilizing chondrocytes, such as ACI, are limited by the size of defects (2–10 cm^2^ diameter. Large defects require more cells; a cell density of 10,000 cells per micro liter (µL) is required in ACI and this is limited due to the need for large quantities of autologous tissue harvested from healthy regions of joints [19]. The chondrocytes are harvested from cartilage tissue explanted from non-weight bearing regions of the joint. The quantity of tissue that can be explanted is limited due to the risk of creating secondary critically sized defects as well as donor site morbidity.

Another drawback associated with the use of chondrocytes is the requirement for monolayer expansion prior to implantation in ACI procedures. This expansion period can be detrimental to the phenotype of chondrocytes and their capacity to synthesize cartilage-like tissue is diminished as a result. Moreover, the lengthy period of expansion is not a financially viable option [60,61]. ACI procedures have also been associated with drawbacks such as leakage of chondrocytes from defect sites as well as inhomogeneous distribution of cells following injection within the defects [62]. Consequently, the use of alternative sources of cells for cartilage defect repair has been widely investigated and the following section will explore some potential cell types for this application.

#### 4.1.2. Mesenchymal Stem Cells

Adult mesenchymal stem cells (MSCs) have been considered as a promising alternative to chondrocytes in the repair of cartilage lesions. This is mainly attributed to their multi-potency (ability to differentiate into a variety of connective tissue cells including cartilage-like cells) as well as their extensive self-renewal capacity [63]. MSCs also secrete a range of trophic factors such as cytokines and growth factors which mediate cellular activity, from differentiation to immunological response [64]. It has been shown that MSCs secrete factors that suppress immunological response* in vitro* through inhibition of TNF-α and IFN-γ secretion [65,66,67]. These cells have an innate capacity to inhibit T-cell proliferation* in vivo* due to their absence of specific surface markers such as CD80 and CD86 that inhibit T-cell activity [68]. This characteristic offers great advantages for the use of MSCs in regenerative medicine and could have significant impact in progression of future TE interventions [69]. MSCs can be derived from a range of sources such as bone marrow, adipose tissue, fat pad as well as synovium tissue. The following section will explore these distinct sources of MSCs and their advantages and disadvantages are listed in (Table 1).

**Table 1:** Advantages and disadvantages of mesenchymal stem cells (MSCs) from various sources.

MSC type	Advantages	Disadvantages
Bone marrow stem cells [70,71]	Easily isolated from bone marrow High chondrogenic potential Broadly characterized and investigated Homogeneous population	Extracting bone marrow is a very painful and invasive procedure Low yield (approximate 1 in 1 × 10^5^ cells in the marrow) Decline in proliferative and differentiation capacity with age
Adipose-derived stem cells [72]	Abundance of tissue High yield (approximate 5000 stem cells per gram of aspirate) Low donor tissue morbidity	Inhomogeneous cell population
Infrapatellar fat pad-derived stem cells [73]	High chondrogenic potential Low donor site morbidity	Limited source of tissue
Synovium-derived stem cells [74]	High yield High proliferative rate High chondrogenic potential	Limited source of tissue

##### Bone Marrow-Derived Stem Cells

The bone marrow contains multipotent MSCs that demonstrate a capacity to differentiate into different tissue types including cartilage [75]. It is one of the main sources of progenitor cells that are used clinically and they have been well characterized and possess surface markers specific to MSCs, such as CD44, CD90 and CD105. The MSCs derived from the bone marrow in humans are mainly isolated from the iliac crest. The quantity of MSCs in the bone marrow has been shown to decrease with age from infancy to adulthood. A newly born child has approximately 1 MSC per 10,000 cells whereas a 50 year old adult possesses approximately 1 MSC per 400,000 cells in the marrow [64]. Their use in cartilage TE has been extensive both* in vitro* and* in vivo*. Examples include bone marrow derived MSCs cultured as micromass pellets or on three-dimensional systems which have shown potential to differentiate down a chondrocytic lineage [63,76,77].

##### Adipose-Derived Stem Cells

Adipose-derived stem cells (ADSCs) are isolated from lipoaspirates and have been shown to be multi-potent with a capacity to differentiation into cartilage-like cells* in vitro* [78,79]. In addition, these cells have been employed* in vivo* and have the potential to synthesize cartilage matrix molecules [80]. A study investigating the characteristics of these cells demonstrated that they possess surface markers similar to those of other MSCs. Some of these markers include CD44, CD105 and CD166, indicating their true mesenchymal stem-ness [81].

##### Infrapatellar Fat Pad-Derived Stem Cells

The fat pad in joints has recently been identified as a source of progenitor cells giving a higher yield of stem cells without the pain associated with their extraction from tissue, unlike other MSC sources [73,82]. These cells have also been shown to display multipotent characteristics with surface markers such as CD44, CD90 and CD105 which are indicative of MSCs. As a result, infrapatellar fat pad-derived stem cells (IPFSCs) have been recently shown potential in cartilage TE and have shown the capacity to produce cartilage-like matrix [83,84]. Thus, this source of cells is a potentially viable option for application in cartilage defect repair applications.

##### Synovium-Derived Stem Cells

Synovial tissue has also been identified as a source of progenitor cells with multilineage differentiation. The self-renewal capacity of cells derived from the synovium has been shown to be phenomenal. Synovial membrane-derived stem cells (SMSCs) have been shown to be capable of maintaining a linear growth curve over 30 population doublings [85]. The surface epitopes identified on these cells were also similar to other MSCs [86]. SMSCs have been cultured under chondrogenic conditions extensively and have shown considerable potential to synthesize cartilage matrix [86,87].

##### Comparison of the Chondrogenic Potential of MSCs from Various Sources

With the increasing numbers of MSC sources available, the question arises: which cell source offers the greatest chondrogenic potential? Whilst the ideal cell source has not been identified due to the conflicting outcomes of a number of publications, a few noteworthy studies point towards bone marrow- and synovium-derived MSCs as being superior to other sources with regards to their chondrogenic potential (Table 2). Two studies comparing MSCs derived from synovium, bone marrow, periosteum, adipose and muscle tissues demonstrated the superiority of synovium-derived MSCs with regards to their proliferative capacity and cartilage matrix deposition [74,80]. Another study also showed that bone marrow and synovium-derived MSCs supported significantly higher* in vivo* chondrogenic response than adipose and muscle-derived MSCs [88]. The reason behind the superiority of synovium-derived MSCs over other MSCs is not fully understood; however, various studies have shown that the healing response of the meniscus and articular cartilage tissue may be largely attributed to the synovium tissue [89,90]. Studies have also showed that synovium-derived MSCs have an age-independent proliferative and differentiation capacity whereas bone marrow MSCs are age-dependent [70,85]. It should also be noted that differences in isolation and culture protocols may be the main contributing factor in the differences seen between different studies with regards to the response of stem cells from various sources [91]. Moreover, the homologous use of such cells may also be a driving factor for their enhanced chondrogenic potential.

There is growing evidence that there exists a population of progenitor cells that reside within the articular cartilage [92]. These cells are known to support appositional cartilage growth from the surface. Such chondroprogenitor cells demonstrate a degree of plasticity with potential to form other connective tissue types including bone. However, the chondrogenic potential of these articular progenitor cells may offer an alternative to the different MSC sources. The main disadvantage of this source of cells however, is the need to extract considerable amounts of articular cartilage to obtain a sufficient quantity of cells for expansion and subsequent implantation.

**Table 2:** Comparison of the chondrogenic potential of MSCs derived from various tissue types.

Article	MSC Type Investigated					Outcome
	Adipose	Bone marrow	Muscle	Synovium	Periosteum	
Sakaguchi* et al.*, 2005 [93]	√	√	√	√	√	Synovium-derived MSCs displayed greater chondrogenic response
Yoshimura* et al.*, 2006 [74]	√	√	√	√	√	Synovium-derived MSCs displayed greater chondrogenic response
Koga* et al.*, 2008 [88]	√	√	√	√	–	Bone marrow and synovium-derived MSCs displayed greater chondrogenic response
Havlas* et al.*, 2011 [94]	√	√	–	–	–	No difference between adipose and bone marrow MSC chondrogenic response
Vidal* et al.*, 2008 [95]	√	√	–	–	–	Bone marrow-derived MSCs displayed greater chondrogenic response
Reich* et al.*, 2012 [96]	√	√	–	–	–	Bone marrow-derived MSCs displayed greater chondrogenic response

#### 4.1.3. Co-Culture Systems

Co-culture systems are emerging as novel methods to enhance the differentiation and chondrogenesis of MSCs. The terminal differentiation, hypertrophy and potential mineralization by MSCs in chondrogenic conditions* in vitro* is a major drawback and may alter the full regenerative potential of these cells. This unstable differentiation may progress towards an endochondral ossification pathway [97]. As a result, there is a general requirement to maintain the chondrogenic differentiated state of MSCs. However, the use of co-culture systems offers a solution to such a drawback. There is growing research into the cross-talk between different cell types and how this can enhance their regenerative capacity. Chondrocytes have been shown to secrete parathyroid hormone related protein (PTHrP) when co-cultured with MSCs subsequently inhibiting the hypertrophy of the MSCs [98,99]. Conversely, MSCs have also been shown to enhance the proliferation of chondrocytes as well as maintaining their phenotype as a result of synthesis of trophic factors [100]. In addition, these co-culture systems have been shown to up regulate chondrocyte related gene expression and production of cartilage-like matrix* in vitro* [101]. Therefore, co-culture systems may overcome some of the drawbacks associated with the use of chondrocytes and MSCs independently. One disadvantage of these co-culture systems is the complexity of their use as well as the regulatory hurdles which may hinder progress in cell-based approaches to cartilage defect repair applications.

### 4.2. Advances in Scaffolds for Cartilage Defect Repair

A three-dimensional structure is an important characteristic of TE scaffolds as the scaffold acts as a template to support interaction, proliferation of cells and deposition of ECM. Moreover, this three-dimensional environment should ideally support maintenance of the characteristic cellular phenotype and function. Although the composition and surface chemistry of scaffolds plays a significant role in the behavior of cells, the structural integrity of the scaffold must also be suitable to support the growth of cells. Having the optimal equilibrium between the appropriate composition and structural integrity has proven complex. Ideal characteristics of articular cartilage grafts include biocompatibility, porous nature to support cell and tissue in-growth, suitable surface chemistry to support cell attachment as well as chondro-inductivity [102,103]. Additionally, this ideal biomaterial must be able to sustain such characteristics whilst degrading in a manner that does not hinder further tissue regeneration [104]. Some of these features will be discussed in more detail below.

#### 4.2.1. The Effect of Scaffold Composition on Chondrogenesis

The composition of scaffolds and their associated surface chemistry affects cellular adhesion, morphology and subsequently the type of tissue synthesized [105]. Scaffolds can be generally grouped into two types; synthetic-based and natural-based. Synthetic scaffolds have the advantage of having controllable structural and mechanical properties whereas natural scaffolds have the advantage of greater cellular interaction due to the presence of ligands which may facilitate cell adhesion. These two scaffold types are presented below highlighting the characteristics that favor their biofunctionality.

##### Synthetic Materials Utilized in Cartilage Tissue Engineering

The most commonly used synthetic materials include polycaprolactone (PCL) poly L-lactic acid (PLLA) and polyglycolic acid (PGA) [106,107,108]. An advantage of synthetic materials is that they are pathogen-free and there is low potential for immunological rejection. PCL scaffolds have been used for* in vitro* chondrogenesis with MSCs exhibiting chondrogenic gene expression and matrix deposition [76,109]. The controllable degradation rate of polymeric scaffolds has been taken advantage of in the development of scaffolds for growth factor delivery [110]. These materials can be fabricated into a range of different forms, such as particles, meshes and fibers demonstrating another advantage of polymers. However, the main drawback of synthetic materials is that their products of degradation are highly concentrated acids and particulates that can result in localized inflammation and cell death [111]. Smith and Nephew’s TRUFIT CB^®^ is a licensed and commercially available synthetic bi-layered product that is composed of poly-(DL-lactide-co-glycolide or PDLG) as well as calcium sulphate for chondral and osteochondral tissue repair applications.

##### Naturally-Derived Materials

Various natural materials used in TE are derived from components of the extracellular matrix (ECM), with polypeptides and polysaccharides being the most widely used due to their biodegradability and non-toxic degradation products. Collagen has received the most attention in TE [112,113,114]. This is partly due to its ubiquitous nature as well as its mechanical properties that exceed those of other natural materials. Additionally, collagen can easily be tailored to improve its mechanical properties and degradation rate, such as through the use of cross-linking treatment [115]. Collagen also possesses a number of functional groups along its backbone allowing interaction with other molecules, such as polysaccharides and even protein-based growth factors [33]. While Type I collagen is traditionally used in collagen-based scaffolds for TE, type II collagen, the principal collagen present in articular cartilage, is of particular interest as a material for use in cartilage tissue engineering and repair applications. A number of studies have shown the potential of scaffolds and gels containing collagen type II to initiate and maintain MSC chondrogenesis [116,117].

The biofunctionality and regenerative capacity of collagen scaffolds can be significantly improved by the addition of GAGs [118]. Indeed, scaffolds fabricated from co-polymers of collagen and GAG have been successfully applied* in vitro* and* in vivo* for skin, tendon, nerve and conjunctiva [119,120,121]. In our laboratory, chondroitin sulphate has been utilized to fabricate scaffolds for bone repair applications [122,123]. Chondroitin sulphate has also been used in cartilage defect repair applications in combination with other biomolecules. It has been commonly co-polymerized with collagen and has been shown to enhance chondrocyte proliferation and matrix production* in vitro* [124,125]. Hyaluronic acid, a non-sulphated GAG, is a natural material found natively in articular cartilage tissue and is synthesized as a long macromolecule with a molecular weight of over 1 MDa but still maintains a high level of degradability. Hyaluronic acid is used in the fabrication of a commercially available matrix, HYAFF II^®^ (Fidia Advanced Biopolymers, Italy) for osteochondral application. HYAFF II^®^ is a derivative of hyaluronic acid formed by esterification of carboxyl groups of the glucuronic acid with benzyl alcohol. This material has been shown to support cellular compatibility and enhancement of chondrogenesis* in vitro* by up-regulation of chondrogenic gene expression as well as collagen type II and aggrecan deposition [126]. Hyalograft C^®^ was subsequently developed from the HYAFF II^®^ scaffold for MACI applications [58,127]. The use of other natural materials such as chitosan, which is derived from the shells of crustaceans, for cartilage defect repair application has been realized. BST-CarGel which is marketed by Piramal Healthcare is a hydrogel-based material composed of chitosan [32]. It is used in combination with bone marrow stimulation and has recently been approved for clinical use.

#### 4.2.2. The Effect of Scaffold Geometry on Chondrogenesis

**Figure 3:**
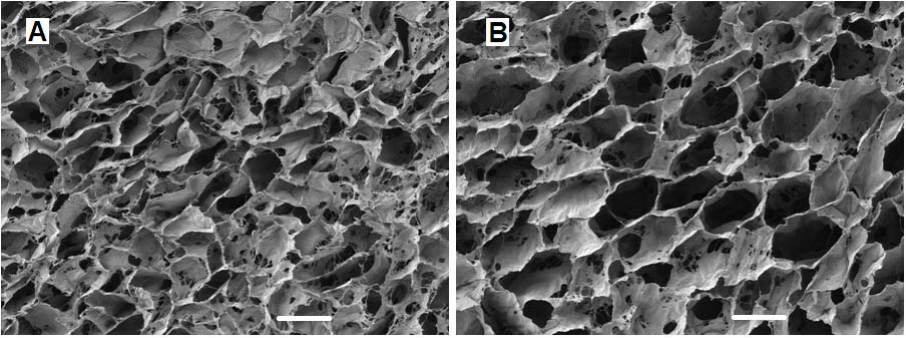
Scanning electron microscope images of collagen-glycosaminoglycan scaffolds with two distinct mean pore sizes; 94 µm (**A**); 130 µm (**B**). The scale bar represents 100 µm.

The geometry and microarchitecture of scaffolds are key factors that determine adhesion and migration of cells as well as the retention of synthesized matrix [109,122,128]. Pore size and porosity are important characteristics of scaffolds that can affect cell infiltration and matrix production [129]. It is now known that scaffold pores need to be large enough to allow cell migration, where cells eventually become bound to ligands presented by the scaffold, but also small enough to establish a sufficiently high specific surface area for cell adhesion [122]. Whilst it is clear that cell adhesion is affected by scaffold mean pore size, the behavior of different cell types may be influenced by microarchitecture. Cells may require distinct scaffold geometries for adhesion, migration and proliferation due to their varying morphologies and sizes, suggesting that from a scaffold development perspective, pore size is cell-type specific [130,131]. Therefore, further investigation and understanding of mechanisms by which biomaterial architecture affects cell-scaffold interaction and subsequent cartilage tissue formation still remains of significant importance. Using a lyophilisation process, collagen-based scaffolds can be fabricated in our laboratory with a range of distinct mean pore sizes (Figure 3). A previously published study carried out in our laboratory has shown that larger mean pore sizes encourage MC3T3 cell (pre-osteoblast-like cell line) attachment, proliferation and migration [128]. Furthermore, unpublished work from our group has also shown a similar trend of increasing cell attachment and proliferation with increasing mean pore size with a different cell type (MSCs). We therefore hypothesize that a scaffold mean pore size range of approximately 300–350 μm diameter can support enhanced cell infiltration and matrix deposition. Although both MSCs and chondrocytes are much smaller in diameter (approximately 5–15 µm), their attachment and migration on scaffolds can be largely affected by the mean pore size. We recently observed encapsulation of cells on the periphery of collagen-glycosaminoglycan scaffolds with a mean pore size of less than 100 µm whilst scaffolds with mean pore sizes greater than 300 µm encouraged improved cell infiltration towards the center.

#### 4.2.3. The Effect of Scaffold Mechanical Properties on Cell Behavior

The mechanical properties of scaffolds for cartilage TE play a significant role in the regeneration of damaged tissue. Scaffolds must be able to support cell growth and still possess the durability to remain uncompromised by the normal functioning of the joint until full regeneration occurs. Articular cartilage tissue within joints experiences high compressive stress throughout its normal functioning. Therefore, a scaffold designed for cartilage tissue repair must be able to withstand at a minimum, loads applied as part of the tissue regeneration process. On a cellular level, the mechanical properties of scaffolds affect the differentiation of MSCs. It is becoming clear that the mechanical properties of a substrate greatly affect the lineage that undifferentiated cells follow. Indeed, it has been shown that the substrate stiffness can direct MSC differentiation towards specific lineages [132,133]. Engler* et al.* (2006) [132] showed that on gels with low stiffness (0.1–1 kPa), MSCs expressed high levels of neurogenic transcriptional factors (NGF, GDNF) as well as neurogenic-specific cytoskeletal factors. On gels investigated with intermediate stiffness (11 kPa), MSCs expressed the highest level of myogenic transcriptional factors (Pax3, MyoD1). However, on the stiffest gels investigated (34 kPa), the seeded MSCs expressed the highest level of osteogenic factors (BMP1, BMP4, CBFα1). Murphy* et al.* (2012) [133] also showed that the scaffolds with the stiffest compressive modulus also led to early stage expression of osteogenic markers, whilst the most compliant scaffolds supported early stage chondrogenic differentiation in the absence of differentiation factors. Therefore, the use of the mechanical properties of scaffolds to direct and enhance the differentiation of MSCs demonstrates the importance of such a characteristic in the design and development of advanced tissue engineering strategies for cartilage defect repair.

Studies on stem cell differentiation have traditionally employed growth factors, however, more recently a number of studies have demonstrated strong interaction between the mechanical properties of substrates (utilizing hydrogels and two-dimensional culture systems) and subsequent cellular differentiation. MSCs are capable of demonstrating lineage-specific differentiation when cultured on substrates with stiffness that closely matches that of the analogous native tissue [134]. The Young’s modulus, often referred to as elasticity or stiffness is an intrinsic ECM characteristic that has considerable effect on cell migration, morphology and proliferation [113,135]. The mechanism by which cells respond by translating these intrinsic mechanical signals into a cascade of biochemical events resulting in gene expression is not fully understood. However, it is becoming more apparent that key roles in molecular pathways are played by adhesion complexes and the actin-myosin cytoskeleton [136].

**Figure 4:**
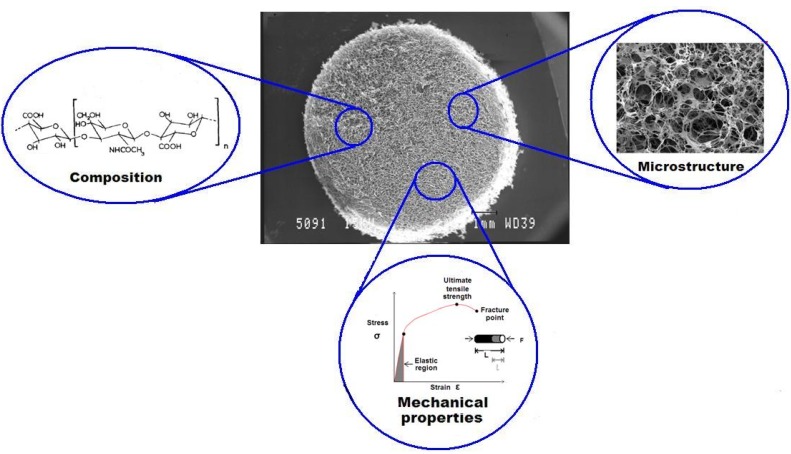
An illustration of three scaffold characteristics that have a fundamental role in the response of cells. One important property is the microstructure of scaffolds that may influence cell infiltration (central scaffold SEM image reprinted with permission from [38], copyright (2012) Elsevier). Magnified SEM image shows the microstructure of a collagen-based scaffold. The chemical composition is another important property that can alter cell-scaffold adhesion. The chemical structure represents a hyaluronic acid disaccharide unit, n~5000 (Reprinted with permission from [138], copyright 1972 Nature Publishing Group). The mechanical property of the scaffold also plays an important role in cell response by influencing cell proliferation, differentiation and migration (the graph shows a typical stress-strain curve of a ductile material).

While the composition of scaffolds is fundamental for cell adhesion through specific ligands on the scaffold surface, the scaffold stiffness determines how the cells ‘feel’ their surrounding matrix [133]. Cells rely on their substrates by pulling and “feeling” the resistance to deformation which provides feedback through mechano-sensitive molecules [137]. These intrinsic mechano-sensitive molecules play a significant role in cartilage development and may help guide cells as they mature and assemble during repair of damaged tissue. The ideal combination and interplay between these three properties is complex, however, an understanding of their role through identification of expression markers is imperative and is fundamental in the development of scaffolds for chondrogenesis (Figure 4).

### 4.3. Biomolecules for Cartilage Defect Repair

Small chondral lesions have been the target of numerous scaffold-based (biomolecule-free) systems developed in TE. However for large defects, localized control over cell and tissue response may be required to enhance the healing response. Subsequently, the use of cytokines, hormones and growth factors has been adopted in the field of TE to target such large defects. Articular cartilage is inherently exposed to a range of biochemical and biophysical stimuli physiologically that affect its homeostasis and capacity for regeneration. This section describes some of the key biochemical factors that play a significant role in cartilage defect repair. In particular, the use of different biomolecules such as growth factor as well as gene therapy approaches which have been used to enhance stem-cell mediated chondrogenesis is highlighted in this section.

#### 4.3.1. Growth Factors

Due to the drawbacks associated with the use of chondrocytes such as poor motility and matrix deposition, researchers have employed growth factors to overcome such limitations. The potential therapeutic application of growth factors has been identified in the field of TE and there is an exponential increase in research in this prospective area. Soluble growth factors used for* in vitro* chondrogenesis studies include transforming growth factor beta (TGF-β), basic fibroblast growth factor (bFGF) and insulin growth factor (IGF) [139,140,141]. Bone morphogenetic proteins (BMPs) are the most widely available growth factors clinically. BMP-4, -6, and -7 have been shown to promote maintenance of a chondrogenic phenotype and up-regulation of cartilage matrix synthesis [97,142]. Insulin-like growth factor-1 (IGF-1) is the main anabolic growth factor of articular cartilage and stored in the ECM bound to proteoglycans via IGF-1 binding proteins. With increasing catabolic activity, the proteolysis of binding proteins also increases and controls the release of IGF-1 [143].

Fibroblast growth factor (FGF) is a widely investigated growth factor with 22 different forms identified so far. Cells expanded in the presence of FGF-2 demonstrate greater chondrogenic capacity when transferred to three-dimensional culture [141,144]. The maintenance of a chondrocytic phenotype is important for* in vitro* culture in order to prevent dedifferentiation, common with chondrocytes in two-dimensional systems and in addition, to sustain matrix deposition over a prolonged period of time. From an* in vivo* perspective, there are some drawbacks associated with the use of bFGF including its non-specificity. In addition, bFGF alone cannot elicit cartilage tissue regeneration demonstrating a requirement for a secondary factor to support enhanced chondrogenesis.

TGF-β is the most commonly used growth factor for* in vitro* chondrogenesis [63,145]. In particular, TGF-β and its isoforms (-1, -2 and -3) have been shown to evoke a chondrogenic response in progenitor cells including MSCs. It is a multi-functional growth factor that modulates a range of activities, such as control of proliferation and differentiation in addition to maintenance of a chondrocytic phenotype of differentiated cells. TGF-β is involved in cartilage homeostasis and is secreted in an inactive form bound to a peptide from which it dissociates before becoming active and bound to its target receptor. The disadvantages of TGF-β are that it is relatively expensive and the potential of chondrocyte hypertrophy through the use of sustained high quantities of such a growth factor poses a major problem with regards to subsequent mineral deposition. In this regard, a number of studies have shown that a combination of TGF-β with other growth factors leads to greater chondrogenic response than TGF-β alone [139,146]. Owing to the chondro-inductivity of TGF-β, the potential therapeutic application of this growth factor in cartilage defect repair is a significant area that requires investigation.

Other growth factors that have shown potential for use in cartilage tissue engineering more recently include parathyroid hormone related peptide (PTHrP) and platelet-derived growth factor (PDGF). PTHrP has been used for MSC and chondrocyte culture, and has exhibited delayed progression towards hypertrophy and ossification [147]. Similarly to PTHrP, PDGF has been shown to enhance cartilage matrix production by chondrocytes whilst preventing them from advancing towards an endochondral maturation pathway through inhibition of alkaline phosphatase activity [148]. PDGF has also been shown to up-regulate the proliferative capacity of chondrocytes* in vitro* [149].

A number of commercially available products that are based on growth factors have been approved for clinical use demonstrating the promise of such an approach for cartilage defect repair. Stryker’s OP-1^®^ and Medtronic’s INFUSE^®^ both for bone tissue application were one of the first growth factor-based products available clinically. Their approval demonstrates a significant step for the future of this field with regards to regulatory barriers being overcome.

One major disadvantage of using growth factors for tissue regeneration is the potential for ectopic tissue formation* in vivo*. A classical example is BMP-2, which was approved by the FDA for bone tissue regeneration was found to elicit ectopic bone formation. Another disadvantage, the relatively short half-lives and short-range diffusion of growth factors suggests that a carrier system is required to enhance the action of such proteins for tissue regeneration. Therefore, the control of delivery and dosage of growth factors needs to be considered highly prior to their application* in vivo*.

#### 4.3.2. The Use of Scaffolds for Delivery of Growth Factors

The drawbacks associated with a number of growth factor-based products including uncontrolled protein delivery demonstrate a requirement for improved carrier systems. High doses of growth factors* in vivo* may be associated with tumorogenesis as well as ectopic tissue formation. Consequently, there is an inherent need for optimal delivery systems that ensure targeted spatial-temporal release of the therapeutic agent on demand. Scaffolds have the potential to act as carriers for therapeutic agents. The successful immobilization of growth factors within scaffolds demonstrates even further the potential of using biomaterials to regulate cell behavior and subsequently modulate a repair response.

A number of studies have taken advantage of the degradation properties of scaffolds to control precisely the signaling of growth factors in a local area. The release of the desired growth factor from the biomaterial on demand is the main goal of this area in TE. This can be achieved through response and interaction of these delivery systems to stimuli such as pH, temperature and enzymatic activity [150,151]. A wide range of growth factors have been used within delivery systems and in particular, attempts have been made to incorporate TGF-β into biomaterials and scaffolds to improve their chondro-inductive nature [145,152].

The incorporation of growth factors onto scaffolds either chemically through immobilization or physically through encapsulation has been widely investigated in TE [150]. This has been achieved either through covalent conjugation of the growth factors onto the biomaterial or through secondary interactions between the growth factor and the biomaterial. With non-covalent incorporation, the absorption of the growth factor exploits direct charge-to-charge interaction with the biomaterial [153]. Secondary incorporation may involve the addition of micro-particles that are loaded with the desired growth factor, into the biomaterial [154].

**Figure 5:**
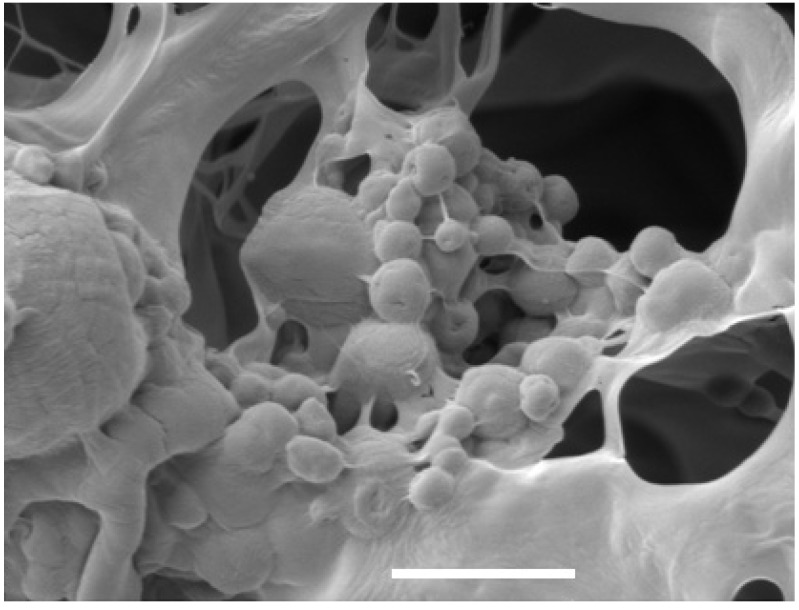
Scanning electron microscope image of a collagen scaffold incorporated with alginate micro-particles. The micro-particles can be seen adhered to the struts of the scaffolds. The scale bar represents a 5 µm length.

Micro-particles are becoming increasingly used for delivery of growth factors such as TGF-β in order to provide greater control over the release kinetics of the growth factor. Micro-particles developed from PLGA, chitosan, alginate and gelatin have been widely characterized and have shown great potential to control delivery of growth factors [152,155,156]. Within our laboratory, we have fabricated micro-particles from a range of materials including alginate as shown in (Figure 5). These particles can be used as carriers of growth factors and incorporated within scaffolds to enhance the stability of the biomolecules. A major disadvantage of such micro-particle-based delivery systems is the low efficiency of encapsulation of the growth factors.

### 4.4. Gene Therapy in Tissue Engineering

Gene therapy has the potential to overcome the problems associated with localized delivery of growth factors. Such problems include the short half-life of growth factors, the potential requirement for repeated application as well as poor delivery of the agent resulting in growth factor diffusion away from desired area [157]. By using gene transfer to deliver a therapeutic protein to a target cell or tissue, the cells essentially act as “factories” that synthesize the protein and induce that cell or tissue to engage in repair or regeneration and guide healing. This approach has been proposed to bear great significance in the field of cartilage defect repair [158]. Recent advances in this field have led to the use of plasmid DNA containing a reporter gene that can stimulate a desired gene expression [159]. More recently, scaffolds have been developed and used as carrier systems for plasmid DNA to develop what are now termed as “gene activated matrices” (GAMs) [160]. GAMs are biodegradable scaffolds containing vectors with genes that can elicit the desired gene expression. Consequently, the GAMs act as delivery vehicles for genes whilst also supporting matrix retention [161]. This offers great potential in the field of TE especially for cartilage defect repair application. These GAMs have been employed in cartilage TE by incorporating plasmid DNA containing reporter genes that can elicit a chondrogenic response of seeded cells [162,163].

The introduction of the desired gene together with the encoding DNA plasmid into the target cell is facilitated by either viral or non-viral vectors [164,165,166]. Whilst viral vectors have been shown to promote higher transfection efficiencies, the concerns associated with immunogenicity has warranted more emphasis on non-viral vectors. A study using a collagen-glycosaminoglycan (CG) scaffold as a carrier for non-viral vectors containing IGF-1 recombinant protein showed that seeded chondrocytes synthesized significantly higher cartilage-like matrix than control scaffolds. Moreover, the release kinetics of IGF-1 could be further controlled by the use of cross-linking treatment methods [167].

Some elegant studies involving dual axis therapy have showed great potential in cartilage defect repair applications [168,169]. These studies used a combination of anabolic (IGF-1) and catabolic (IL-1 antagonist) proteins to regulate tissue homeostasis using gene therapy. The catabolic proteins inhibit expression of genes related to catabolic tissue response whilst anabolic proteins stimulate matrix production. Other studies using gene therapy for cartilage repair applications have utilized growth factors such as TGF-β and BMPs [170,171].

For a number of years, major concerns have existed about the use of gene therapy in clinical applications and major regulatory hurdles exist with their introduction in regenerative medicine. However, the approval of the first gene therapy product, Glybera (UniQure) for the treatment of lipoprotein lipase deficiency, by the European Union in November 2012 marks a significant milestone for the scientific community in general and specifically for the TE field as it opens a gateway for more research in this novel area. It provides potential for more funding from grant funding bodies and venture capitalists to be directed towards these areas and optimism for researchers to see their work proceed from bench-top towards commercialization.

## 5. Concluding Remarks

Tissue engineering (TE) has witnessed significant advances as a result of the collaboration between researchers from cell biology, biomaterial science and engineering. The latest research in the field of cartilage TE suggests a shift away from the use of chondrocytes to multipotent mesenchymal stem cells (MSCs). The identification of MSCs within different sources such as synovial and adipose tissue has offered a solution to the drawbacks associated with the use of chondrocytes. In addition, their immunosuppressive characteristics offer great benefits for researchers carrying out* in vivo* studies. The ever growing use of biomimetic approaches in designing biomaterials for cartilage defect repair demonstrates significant advances in this field. Moreover, the incorporation of recombinant growth factors to further improve the chondro-inductivity and subsequent regenerative capacity of scaffolds may play a major role in development of constructs for large chondral defects. The emergence of gene therapy in TE which has been identified to offer superior localized control over cell and tissue response demonstrates significant promise in the development of advanced TE strategies for cartilage defect repair. Accordingly, such progress demonstrates affirmative prospects for the field of TE and offers a promising alternative to the current solutions to the problem of articular cartilage defect repair.
